# An efficient strategy for cell-based antibody library selection using an integrated vector system

**DOI:** 10.1186/1472-6750-12-62

**Published:** 2012-09-18

**Authors:** Hyerim Yoon, Jin Myung Song, Chun Jeih Ryu, Yeon-Gu Kim, Eun Kyo Lee, Sunghyun Kang, Sang Jick Kim

**Affiliations:** 1 Immunotherapy Research Center, Korea Research Institute of Bioscience and Biotechnology, 111 Gwahangno, Yuseong-gu, Daejon, 305-806, Republic of Korea; 2 Institute of Bioscience, Department of Bioscience and Biotechnology, Sejong University, 98 Gunja-dong, Gwangjin-gu, Seoul, 143-747, Republic of Korea

**Keywords:** Phage display, Antibody library, CD9, Cell panning, scFv-Fc

## Abstract

**Background:**

Cell panning of phage-displayed antibody library is a powerful tool for the development of therapeutic and imaging agents since disease-related cell surface proteins in native complex conformation can be directly targeted. Here, we employed a strategy taking advantage of an integrated vector system which allows rapid conversion of scFv-displaying phage into scFv-Fc format for efficient cell-based scFv library selection on a tetraspanin protein, CD9.

**Results:**

A mouse scFv library constructed by using a phagemid vector, pDR-D1 was subjected to cell panning against stable CD9 transfectant, and the scFv repertoire from the enriched phage pool was directly transferred to a mammalian cassette vector, pDR-OriP-Fc1. The resulting constructs enabled transient expression of enough amounts of scFv-Fcs in HEK293E cells, and flow cytometric screening of binders for CD9 transfectant could be performed simply by using the culture supernatants. All three clones selected from the screening showed correct CD9-specificity. They could immunoprecipitate CD9 molecules out of the transfectant cell lysate and correctly stain endogenous CD9 expression on cancer cell membrane. Furthermore, competition assay with a known anti-CD9 monoclonal antibody (mAb) suggested that the binding epitopes of some of them overlap with that of the mAb which resides within the large extracellular loop of CD9.

**Conclusions:**

This study demonstrates that scFv-Fc from mammalian transient expression can be chosen as a reliable format for rapid screening and validation in cell-based scFv library selection, and the strategy described here will be applicable to efficient discovery of antibodies to diverse cell-surface targets.

## Background

Phage display of antibody fragments is now well established technology and has enabled the construction and selection of a large size of antibody library *in vitro*[1,2]. Selection of phage library includes panning procedure for enrichment of target-specific phage and subsequent screening procedure for individual phage clones. For the evaluation of candidate clones, antibody fragments in the form of scFv or Fab can usually be expressed in enough quantity by bacterial cells. Though bacterial expression is sufficient for most purposes, some sensitive biological assays as well as *in vivo* validation prefer mammalian expression of antibody. Several reports has thus described construction of cassette-type vectors for rapid conversion of phage-displayed antibody fragments into whole IgG or scFv-Fc format to accelerate the validation process that is done under *in vivo* conditions closely mimicking those expected to occur with therapeutics and imaging agents [3-5].

For the development of therapeutic or imaging agents, cell surface antigens are attractive targets. Cell panning procedure that allows selection of phage-displayed antibody library directly on intact cells has been employed to target the antigens in their native conformation at the surface of cells [6-10]. The procedure can overcome the limitations of the conventional selection procedure using purified recombinant antigens immobilized on artificial surfaces. In fact, some cell surface proteins cannot be expressed in recombinant forms that retain their native conformation, and antibodies selected using the recombinant proteins may not bind to original proteins on cell surface. Furthermore, the procedure gives chances to target novel epitope space created by disease-related overexpression or modification of cell surface proteins.

CD9 is a cell surface glycoprotein that belongs to the tetraspanin family containing four transmembrane domains and two extracellular loops [11]. Its expression has been recently reported to be related to some cancers and proposed to be a potential therapeutic target [12-15]. In this study, we aimed to generate antibodies recognizing CD9 on the cell surface in its native conformation. For this purpose, stable transfectant expressing CD9 has been constructed and used for entire process of panning of phage library and subsequent screening and characterization of individual antibody clones.

To facilitate the whole cell-based screening and characterization, we took advantage of an integrated vector system which allows direct conversion of scFv phage into scFv-Fc format [16]. After cell panning on the CD transfectant, the enriched scFv repertoire in phagemid vector, pDR-D1 was transferred into mammalian cassette vector, pDR-OriP-Fc1 simply by cut and paste restriction fragment cloning. Enough amount of scFv-Fc could be obtained from transient expression by using the resulting constructs in HEK293E cells, which enabled rapid identification and characterization of specific binders to cell surface CD9 using flow cytometry, immunoprecipitation and immunofluorescence confocal microscopy. The results demonstrate feasibility of the strategy using the integrated vector system that allows use of scFv-Fc as a reliable format for rapid cell-based antibody screening and validation.

## Results

### Design features of the integrated vector system

**Figure 1:**
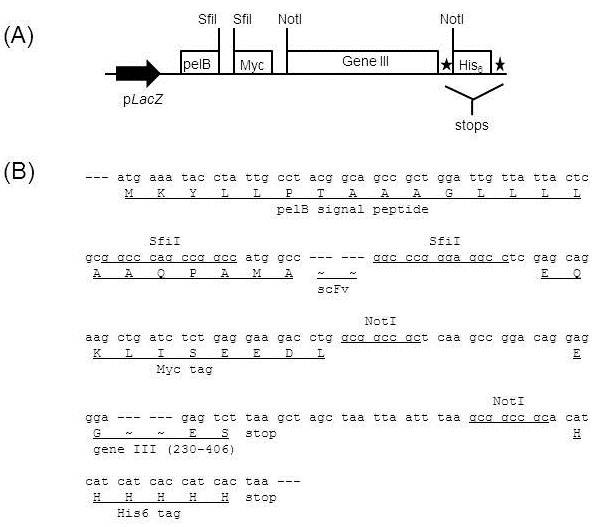
**Schematic representation (A) and sequences (B) of major components of phagemid vector, pDR-D1 for phage display.** The vector is derived from pComb3H with some modifications which result in *Sfi*I-*Sfi*I cassette sequences for scFv cloning followed by *pelB* signal sequences and gene III sequences removable by *Not*I-*Not*I between two tags, Myc and hexahistidine (His6) tags. The expression unit is under the control of the LacZ promoter (p*LacZ*).

**Figure 2:**
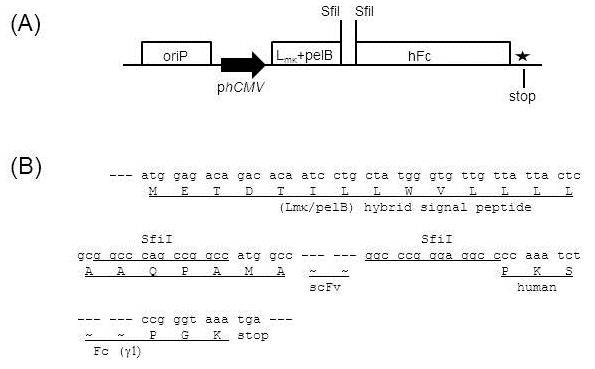
**Schematic representation (A) and sequences (B) of major components of mammalian cassette vector, pDR-OriP-Fc1 for transient expression of scFv-Fc.** The vector is derived from pcDNA3.1 with some modifications which result in the same *Sfi*I-*Sfi*I cassette sequences as pDR-D1 for scFv cloning followed by hybrid signal sequences composed of mouse kappa leader (Lmκ) and *pelB* signal sequences. Human Fcγ1 (hFc) sequences and hinge region are followed by the cloning site to allow in-frame fusion of scFv and hFc. The scFv-Fc expression unit is under the control of the human cytomegalovirus promoter (p*hCMV*). oriP sequences are also included to mediate episomal amplification and maintenance of the transfected episomal DNA in 293E cells.

Here we used two vectors, pDR-D1 (Figure 1A) for phage display of scFv and pDR-OriP-Fc1 (Figure 2A) for mammalian expression of scFv-Fc. They are designed to allow rapid shuttling of scFv inserts, and the sequences of scFv inserts in pDR-D1 can be directly transferred into pDR-OriP-Fc1 simply by cut and paste restriction fragment cloning without PCR-amplification step. Detailed sequences show design features of the integrated vector system (Figure 1B and Figure 2B).

pDR-D1 includes *pelB* signal sequences for periplasmic expression in *E. coli* and two *Sfi*I restriction sites (5’-GGCCNNNNNGGCC-3’) for the cloning of scFv (Figure 1B). The two *Sfi*I sites have different NNNNN sequences, which results in two different sticky ends allowing a directional cloning [17]. The sequences for myc tag are followed by *Sfi*I sites for the detection of scFv expression. Also, pDR-D1 includes gene III sequences (aa 230–406) for the display of scFv. The gene III sequences are designed to be removable by two *Not*I restriction sites for soluble expression of scFv. The sequences for His6 tag are designed to be attached to those for myc tag after removing gene III sequences for the detection and purification of scFv.

For in-frame transfer of scFv from pDR-D1, pDR-OriP-Fc1 was designed to have hybrid signal sequences (Figure 2B). The initial half sequences (METDTILLWV) are derived from mouse kappa leader sequences, and the last half sequences (LLLLAAQPAMA) from *pelB* leader sequences. The derived hybrid signal peptide was proved to be functional in mammalian cells before [5,16]. The resulting construct has the same *Sfi*I sites as those in pDR-D1 and, even unknown sequences derived from pDR-D1 based library can be directly cloned into pDR-OriP-Fc1. The cloned scFv is designed to be fused to human Fcγ1 through the hinge region which results in Fc-fused dimer forms when expressed in mammalian cells. pDR-OriP-Fc1 also includes OriP sequences which allows enhanced and prolonged protein expression in 293E cells containing EBNA-1 [18].

### Selection of antibodies recognizing CD9 on cell surface

A naïve mouse scFv library with an estimated diversity of 2 × 10^9^ was prepared by cloning antibody repertoire derived from mouse splenocytes into pDR-D1 as described in Materials and Methods. Integrity of the library was confirmed by DNA sequencing of scFv inserts from randomly picked library clones. Library phages were rescued using VCSM13 helper phage and confirmed for scFv display on phage surface using western blot (Data not shown).

To select antibodies against native form of CD9 on cell surface, cell panning was performed using a stable CD9 transfectant, HEK293-CD9. The library phages were pre-cleared by subtraction panning with parental HEK293 to deplete non-specific binding phages. The pre-cleared phages were incubated with HEK293-CD9 cells, and bound phages were recovered and amplified for next round of panning. Three rounds of panning were performed, and enrichment of HEK293-CD9 binders was monitored by titering the number of phage recovered after panning. Phage recovery rate (output/input ratio) after the 3^rd^ round was about 200-fold higher than that after the 1^st^ round (Table 1), which indicated successful enrichment of HEK293-CD9 binders.

**Table 1:** Cell panning of scFv library on HEK293-CD9 in three rounds

	**1st round**	**2nd round**	**3rd round**
Phage input (cfu^a^)	1 × 10^13^	3.16 × 10^11^	2.08 × 10^11^
Phage output (cfu)	1.48 × 10^5^	7.40 × 10^4^	6.56 × 10^5^
Recovery rate^b^	1.48 × 10^-8^	2.34 × 10^-7^	3.15 × 10^-6^

^a^ cfu: colony forming unit.

^b^ Recovery rate: output/input ratio used to determine the phage recovery rate of each round.

**Figure 3:**
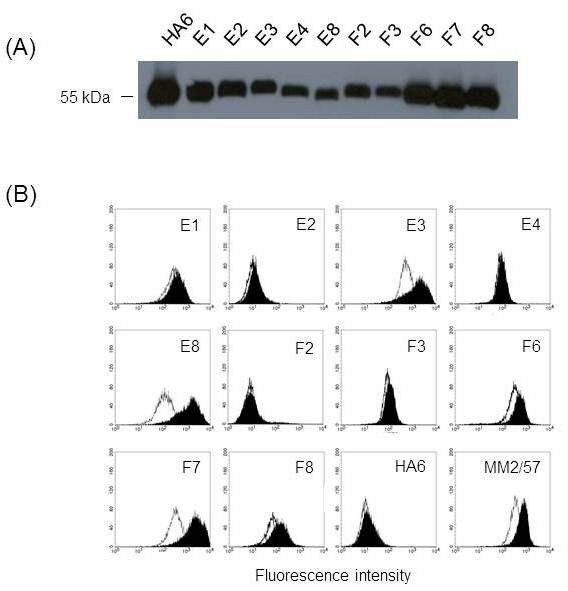
**Screening of scFv-Fcs binding to cell surface CD9.** (**A**) Western blot analysis of scFv-Fc expression. HRP-conjugated rabbit anti-human IgG (Fc-specific) antibody was used to detect scFv-Fc in culture supernatant. (**B**) Flow cytometric analysis of scFv-Fc binding. Culture supernatants containing scFv-Fcs were analyzed for the ability to bind HEK293 (open histogram) and HEK293-CD9 (shaded histogram) by flow cytometry. HA6 represents an scFv-Fc recognizing HAV used as a negative control, and MM2/57 represents an anti-CD9 mAb used as a positive control. The binding of scFv-Fc and MM2/57 was detected by FITC-conjugated rabbit anti-human IgG (Fc-specific) antibody and FITC-conjugated goat anti-mouse IgG antibody, respectively.

For reliable screening of individual antibody clones, scFv-Fc format was chosen instead of phage format. The use of mammalian cassette vector, pDR-OriP-Fc1 allowed the rapid conversion as described above. The scFv repertoire in the phage pool enriched after 3^rd^ round of panning was directly transferred to mammalian cassette vector for scFv-Fc expression by cloning scFv inserts extracted from phagemid DNA prepared after 3^rd^ round of panning into pDR-Orip-Fc1. Twenty clones were randomly selected and subjected to DNA sequencing. Ten unique scFv sequences were identified, and the vectors harboring the scFv sequences were transfected into 293E cells for transient expression. The culture supernatant was confirmed for scFv-Fc expression by detection of 55 kDa bands in western blot analysis (Figure 3A) and directly used for flow cytometric screening of scFv-Fc for its binding to HEK293-CD9 (Figure 3B). HA6 specific for HAV (Hepatitis A Virus) was used as negative control, and MM2/57, anti-CD9 mAb was used as positive control. Three clones (E3, E8 and F7) were strongly positive for binding to HEK293-CD9 and could differentiate CD9 overexpression like MM2/57. However, the other clones such as E1 were not positive and could not differentiate CD9 overexpression (Figure 3B).

### Characterization of scFv-Fcs

For further characterization, the selected scFv-Fcs were purified from the culture supernatants by using protein G column. From 30 ml of serum-free culture supernatant, 100 ~ 200 μg of purified scFv-Fc could be obtained, and its purity was confirmed by a single 55 kDa band on a 12% SDS-PAGE in reducing condition (Data not shown).

**Figure 4:**
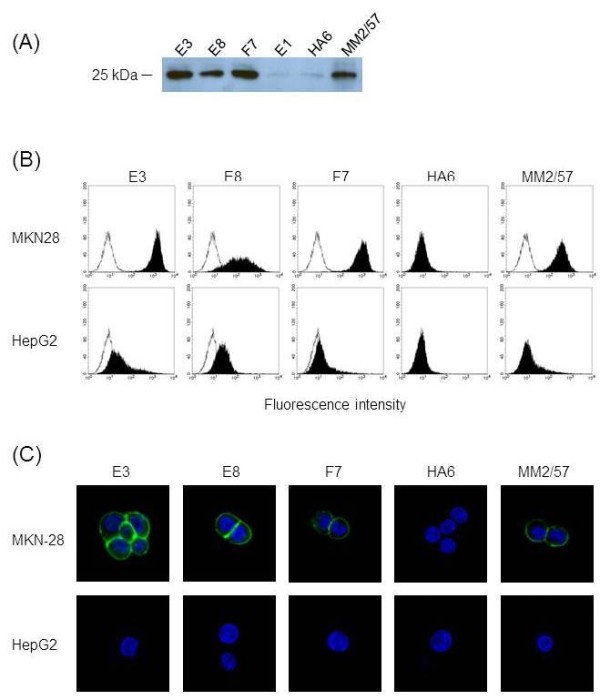
**Characterization of the selected scFv-Fcs.** (**A**) Immunoprecipitation of CD9 molecule by the scFv-Fcs. Cell lysate of HEK293-CD9 was immunoprecipiated by using each antibody indicated on the top of the lane. The immunoprecipitates were resolved by 14% SDS-PAGE and analyzed by immunoblotting with MM2/57. (**B**) Flow cytometric analysis of binding of the scFv-Fcs to endogenous CD9 on human cancer cells. MKN-28 and HepG2 cells were stained with the indicated antibody and the corresponding FITC-conjugated secondary antibody as described in Figure 3. The open histograms in the left represent the data acquired by the FITC-conjugated secondary antibody only. (**C**) Immunolocalization of endogenous CD9 on cancer cells by the scFv-Fcs. MKN-28 and HepG2 cells grown on coverslips were stained with the indicated antibody and the corresponding FITC-conjugated secondary antibody. The cellular nucleus was dyed blue fluorescence with DAPI staining. The stained cells were observed using a confocal microscope (400×).

First, their binding specificity for CD9 was tested by using immunoprecipitation and immunoblotting (Figure 4A). As anticipated, E3, E8 and F7 could pull down about 25 kDa of CD9 molecules from cell lysate of HEK293-CD9, which was recognized by immunobloting with MM2/57. This result confirmed CD9-specificity of the selected antibodies indicating success of the cell-based selection.

Next, we examined whether the selected scFv-Fcs could recognize endogenous CD9 expression on human cancer cells using flow cytometry and immunofluorescence confocal microscopy. Two cancer cell lines were used for comparison. MKN-28, a human gastric cancer cell line is known to overexpress CD9, whereas HepG2, hepatocarcinoma cell line not to express CD9 [19]. As expected, flow cytometric analysis revealed strong binding of all three scFv-Fcs to MKN-28 when compared with their binding to HepG2 (Figure 4B). This strong binding could be confirmed by localizing CD9 staining on MKN-28 with the scFv-Fcs using confocal microscopy (Figure 4C). CD9 staining by the scFv-Fcs and MM2/57 was mainly observed on the cell membrane of MKN-28 but not on that of HepG2 whereas staining by HA6 was barely seen for both cell lines. Hence, all three scFv-Fvs could specifically recognize endogenous CD9 expression on the cell membrane of MKN-28.

**Figure 5:**
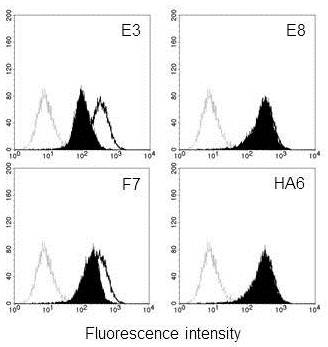
**Flow cytometric analysis of competitive binding of the scFv-Fcs with anti-CD9 mAb, MM2/57.** MM2/57 antibody was incubated with MKN-28 cells in the presence (closed histogram) and absence (open histogram) of the indicated scFv-Fcs. Bound MM2/57 was detected by FITC-conjugated goat anti-mouse IgG antibody. Histograms drawn in dotted line in the far left represent the data acquired by the FITC-conjugated secondary antibody only.

Finally, competition binding experiments using flow cytometry were carried out to test whether the binding epitopes of the selected scFv-Fcs would overlap with that of MM2/57. MM2/57 is known to recognize large extracellular loop (ECL2) of CD9 molecule [20]. As shown in Figure 5, addition of E3 or F7 as competitor inhibited the binding of MM2/57 to MKN-28 cells while addition of E8 did not affect the binding at all like HA6. The data suggest that E3 and F7 share their epitopes with MM2/57 that may be present at ECL2 domain of CD9 molecule.

## Discussion

In this study, we demonstrated an efficient selection of antibodies recognizing cell surface CD9 from phage-displayed scFv library by taking advantage of an integrated vector system. A mouse scFv library was constructed using phagemid vector, pDR-D1, and after cell panning against CD9 transfectant, the enriched scFv repertoire was directly transferred to mammalian cassette vector, pDR-OriP-Fc1. Since the resulting cassette vector constructs enabled transient expression of enough amounts of scFv-Fcs in HEK293E cells, flow cytometric screening of binders for CD9 transfectant could be performed simply by using the culture supernatants (Figure 3). Compared with phage, scFv-Fc format could be preferred choice for the reliable screening of individual clones especially after cell panning procedure. It is known that cell panning procedure usually suffers from high background binding of non-specific phages [21,22]. Likewise, phage screening directly on cell can cause selection of non-specific binders. By adapting scFv-Fc format instead of phage, we wished to discriminate CD9-specific binders from non-specific binders at the screening step. In fact, all three clones selected from FACS screening were found to be CD9-specific (Figure 4A).

Their specificity could be confirmed by recognition of endogenous CD9 expression on cancer cells. It appeared that their binding results were slightly positive even on CD9 negative HepG2 cells by flow cytometry when compared with that of anti-CD9 mAb, MM2/57 (Figure 4B). This might be attributed to their lower specificity. However, all three could correctly localize membrane-bound CD9, and the results were comparable to that of MM2/57 (Figure 4C). Most anti-tetraspanin mAbs including MM2/57 is known to recognize epitopes within ECL2 regions [20]. Our competition data indicated that two of selected scFv-Fcs (E3 and F7) might recognize the immunodominant epitope regions at the ECL2 domain of CD9 which overlap with that of MM2/57 (Figure 5). But the other one, E8 showed different epitope specificity. CD9 like the other tetrapanins can assemble into multimolecular membrane complexes by homo- or hetero-clustering [23-25]. Cell-based selection with intensive screening can give more chances to select rare antibodies recognizing novel epitopes created by such dynamic molecular changes.

Direct selection of phage-displayed antibody library on intact cells is inevitable for some cell surface proteins with complex structural features which make them difficult to be recombinantly expressed in fully native forms. Selection on such recombinant proteins could result in antibodies that failed to correctly recognize their original conformation on cell surface as exemplified previously [10]. Cell-based selection has another advantage over recombinant protein-based selection in that it can give a chance to generate antibodies with novel epitope specificity that can differentiate disease-related cell surface signatures. According to studies on epitope structure of tumor-associated epidermal growth factor receptor (EGFR), EGFR overexpression on cancer cells exposes neo-epitope that is not found in EGFR on normal tissues [26,27]. Besides overexpression, other factors such as aberrant posttranslational modification and multi-molecular clustering can affect conformation of cell surface proteins, which may result in exposure of novel epitope space.

## Conclusions

Cell surface expression of disease-related proteins such as tumor-associated antigens is promising target of antibody development for therapeutic and imaging applications. Cell-based selection using phage-displayed antibody library is a powerful tool for such purpose but needs reliable evaluation of specific binders for efficient selection. As demonstrated in this report, rapid conversion of the scFv phages enriched after cell panning into scFv-Fc format by taking advantage of a mammalian cassette vector will allow efficient development of antibodies for diverse cell surface proteins with complex conformation by accelerating cell-based evaluation steps.

## Methods

### Integrated vector system for antibody display and selection

A phagemid vector, pDR-D1 (Figure 1) was used for cloning and display of antibody repertoire as described previously [16]. The vector includes *pelB* signal sequences, *Sfi*I-*Sfi*I restriction enzyme sites for scFv cloning and sequences for myc tag between lac Z promoter and gene III. Also, hexahistidine (His6) tag is designed to be linked to myc tag after removing gene III using *Not*I-*Not*I restriction enzyme sites for soluble expression and purification of scFv in *E.coli*.

For rapid conversion of phage displayed scFv into Fc-fusion form, a mammalian expression vector, pDR-OriP-Fc1 (Figure 2) was used as described previously [16]. Briefly, the vector was constructed by reengineering pcDNA3.1 (Invitrogen, Carlsbad, CA) to contain human Fcγ1 sequences and hinge region followed by scFv cloning sites as well as OriP sequences. The hybrid signal peptide sequences consisting of leader sequences of kappa chain of mouse IgG and *pelB* were also included upstream of *Sfi*I-*Sfi*I sites for direct cloning of scFv sequences from pDR-D1.

### CD9 expression constructs and cell lines

Human CD9 cDNA was cloned into expression vector, pcDNA3.1/zeo(+) (Invitrogen). Human embryonic kidney-293 (HEK293) cells were transfected with the plasmid using Lipofectamine (Invitrogen). Stable transfectants were selected using 400 μg/ml of zeocine (Invitrogen) in Dubecco’s modified Eagle’s Medium (DMEM, Invitrogen) supplemented with 10% fetal bovine serum (FBS, Invitrogen). A stable transfectant, HEK293-CD9 was used for library panning and selection. Two human cancer cell lines were used for the characterization of CD9-specificity of selected antibodies. Human gastric cancer cell line, MKN-28 was maintained in RPMI-1640 medium (Invitrogen) supplemented with 10% FBS, and human hepatocellular carcinoma cell line, HepG2 in DMEM medium with 10% FBS.

### Library construction and panning

A naïve mouse scFv library was constructed from BALB/c mice (Daehan Biolink Co., Umsong, Korea) by cloning the mouse antibody repertoire into pDR-D1 as described previously [16]. Briefly, a mouse splenocyte suspension was prepared and subjected to total RNA extraction, cDNA synthesis, and PCR amplification of heavy and kappa chain variable regions (VH and VK). The amplified VH and VL containing *Bbs*I site were linked by ligation, and the combined scFv repertoire was subjected to extension PCR with primers containing *Sfi*I site. The amplified scFv inserts and pDR-D1 were digested with *Sfi*I, and, after ligation, the final constructs were electroporated into *E. coli* ER2738 cells. Bacteriophages displaying the scFv repertoire were rescued by the infection of the transformed cells with VCSM13 helper phage (Stratagene, La Jolla, CA).

The rescued phage library was used for cell panning which was performed according to conventional protocols [8,9] with slight modification. The library was pre-incubated with HEK293 cells in PBA (1% bovine serum albumin/0.02% sodium azide/PBS) at 4°C for 1 h. The subtracted phages were recovered by centrifugation and then incubated with HEK293-CD9 cells at 4°C for 1 h. The cells were washed with ice-cold PBA four times, and the bound phages on the cell surface were amplified by infecting ER2738 cells followed by helper phage super-infection. The amplified phages were then subjected to another round of panning.

### Expression and purification of scFv-Fc

After third round of panning, phagemid DNA was extracted from the ER2738 cells infected with the enriched phages. The scFv inserts were cut out of the phagemid by *Sfi*I-digestion and directly cloned into pDR-OriP-Fc1. Twenty clones were randomly selected and subjected DNA sequencing. The nucleotide sequences determined were submitted to IMGT/V-QUEST (http://imgt.cines.fr/vquest) for sequence analysis. As a negative control, scFv against Hepatitis A virus was constructed from VH and VK sequences of HA6 [28] and also cloned into pDR-OriP-Fc1.

The resulting scFv-Fc expression plasmid was introduced into 293E cells (CRL-10852, ATCC) using Lipofectamine. The transfected cells were grown in DMEM containing 10% FBS and subsequently, the media were changed to serum-free media, and the culture supernatant was harvested every 3 day. The culture supernatant was confirmed for scFv-Fc expression by western blot and used for flow cytometric screening of specific binders. For purification of scFv-Fc, the culture supernatant was subjected to affinity chromatography on a Protein G-agarose column (Merck Millipore, Darmstadt, Germany) as described previously [28].

### Flow cytometry

For the screening of scFv-Fcs binding to HEK293-CD9, flow cytometric analysis was performed using the culture supernatant. HEK293 and HEK293-CD9 cells were grown to 70–80% confluence and harvested by trypsinization. About 1 × 10^6^ cells were washed with ice-cold PBS and then blocked with PBA for 10 min at 4 °C. 100 μL of the culture supernatant was added to the cells and incubated for 1–2 h at 4 °C. After washing with PBA, fluorescein isothiocyanate (FITC)-conjugated rabbit anti-human IgG (Fc-fragment specific, Pierce, Rockford, IL) was added to the cells for the detection of bound scFv-Fcs. After 30 min incubation, the cells were washed twice with PBA and resuspended in PBS containing propodium iodide (PI). PI negative cells were gated and analyzed for scFv-Fc binding by using FACSCalibur (BD Bioscience, San Jose, CA) and Cell Quest software (BD Bioscience).

Staining of cancer cell lines with purified scFc-Fv was also performed on MKN-28 and HepG2 cells. Cells grown to 70–80% confluence were harvested and stained as described above. For comparison, a monoclonal antibody (mAb) against CD9, clone MM2/57 (Merck millipore) was used as positive control, and its binding to the cells was detected using FITC-conjugated goat anti-mouse IgG (Pierce).

### Immunoprecipitation and immunoblotting

The binding specificity of scFv-Fc to CD9 was tested by immunoprecipitation and immunoblotting. HEK293-CD9 cells grown to 70-80% confluence were lysed at 4 °C in a lysis buffer (50 mM Tris pH 7.4, 150 mM NaCl, 1% NP-40, 0.5% sodium deoxycholate, 0.1% sodium dodecyl sulfate (SDS), 5 mM sodium fluoride, 1 mM ethylenediaminetetraaceticacid (EDTA), 1 mM ß-glycerophosphate, Xpert Protease Inhibitor Cocktail solution (GenDEPOT, Barker, Tx)). After removing insoluble fraction by centrifugation, the cell lysate was depleted with protein G-agarose for 30 min at 4 °C. The depleted cell lysate was then incubated with scFv-Fc or MM2/57 for 3 h at 4 °C. The immune complexes were captured on protein G-agarose and washed three times with the lysis buffer. After treatment with non-reducing protein loading buffer, the immunoprecipitates were resolved by 14% SDS-PAGE and transferred to PVDF membranes. The membrane was blocked with 4% skim milk in PBS and incubated with MM2/57. After washing with 0.01% Tween20 in PBS, the bound antibody was detected with horseradish peroxidase (HRP)-conjugated anti-mouse IgG (Fc specific, Jackson ImmunoResearch Laboratories, West Grove, PA).

### Immunofluorescence confocal microscopy

Confocal laser scanning microscopy was performed on MKN-28 and HepG2 cells to visualize cell surface binding of scFv-Fcs. The glass coverslips were coated with 0.1% gelatin overnight, washed once with PBS and dried overnight. The cells were seeded on the gelatin-coated coverslips at 2 × 10^5^ cells/slip and allowed to adhere for 48 h at 37 °C. After washing with ice cold PBS twice, the cells were incubated with scFv-Fc or MM2/57 for 2 h at room temperature. Unbound antibody was removed by washing with ice-cold PBS, and the cells were fixed with 4% formalin solution at room temperature for 15 min. After three times washing with ice-cold PBS, bound scFv-Fc and MM2/57 were stained with FITC-conjugated rabbit anti-human IgG and FITC-conjugated goat anti-mouse IgG1, respectively. Finally, coverslips were mounted by applying Vectashield solution (Vector laboratories, Burlingame, CA) and, the stained cells were observed with a confocal laser scanning microscope (Carl zeiss 510 META, Carl Zeiss, Oberkochen, Germany).

## Competing interests

The authors declare that they have no competing interests.

## Authors’ contributions

HY, YGK, EGL, CJR, and SJK designed the research. HY, JMS, and SK performed all the experiments and analyzed the data. HY prepared the initial draft of the manuscript. SJK conceived of the study, coordinated all the components of the project, and prepared the final manuscript. All authors have read and approved the final manuscript.
